# Trends in Swallowing Outcomes Following Deintensified Treatment in Selected p16+ Oropharyngeal Carcinoma

**DOI:** 10.1002/oto2.47

**Published:** 2023-03-24

**Authors:** Esther Lee, Hari Magge, Isabel Park, Leyn Shakhtour, Ning‐Wei Li, Jennifer Schottler, Arjun S. Joshi, Punam G. Thakkar, Joseph F. Goodman

**Affiliations:** ^1^ Division of Otolaryngology–Head and Neck Surgery George Washington University School of Medicine and Health Sciences Washington District of Columbia USA

**Keywords:** MD Anderson Dysphagia Inventory, oropharyngeal squamous cell carcinoma, radiation, swallowing, transoral robotic surgery

## Abstract

**Objective:**

Identify trends in swallowing outcomes in p16+ oropharyngeal squamous cell carcinoma following neoadjuvant chemotherapy+surgery (NAC+S) versus neoadjuvant chemotherapy+surgery+radiation (NAC+S+R).

**Study Design:**

Cohort study.

**Setting:**

Single academic institution.

**Methods:**

Swallowing outcome was measured using a validated questionnaire, MD Anderson Dysphagia Inventory (MDADI). MDADI scores were compared between NAC+S and NAC+S+R groups in short‐term (<1 year), middle‐term (1‐3 years), and long‐term (>3 years). Clinical factors associated with MDADI scores were explored using a linear mixed model. Statistical significance was established at *p* < .05.

**Results:**

Sixty‐seven patients met the inclusion criteria and were divided into 2 groups: NAC+S (57 [85.1%]) and NAC+S+R (10 [14.9%]). All patients had improved MDADI scores in the middle‐term compared to short‐term (NAC+S: score increase = 3.43, *p* = .002; NAC+S+R: score increase = 11.18, *p* = .044), long‐term compared to short‐term (NAC+S: score increase = 6.97, *p* < .001; NAC+S+R: score increase = 20.35, *p* < .001), and long‐term compared to middle‐term (NAC+S: score increase = 3.54, *p* = .043; NAC+S+R: score increase = 9.18, *p* = .026). NAC+S patients had better MDADI scores than NAC+S+R patients at short‐term (83.80 vs 71.26, *p* = .001). There was no significant difference in swallowing function in the middle‐term or long‐term.

**Conclusion:**

Regardless of treatment type, swallowing will likely be improved in the middle‐term and long‐term compared to the short‐term. Patients treated with NAC+S+R will have worse short‐term swallowing function. However, in the middle‐term and long‐term, there is no significant difference in swallowing function between patients treated with NAC+S and NAC+S+R.

Within the last 40 years, the incidence of oropharyngeal squamous cell carcinoma (OPSCC) has remarkably increased in the United States, with an estimated 54,000 new cases and 11,230 deaths in 2022.^1^, ^2^ This rise is largely due to the increase in the incidence of human papillomavirus (HPV)‐positive (HPV+) OPSCC. Typically, HPV+ OPSCC patients tend to be younger and healthier with a good prognosis for survival after treatment compared to HPV‐negative OPSCC.^3^ As such, there has been great interest in de‐escalated treatment for HPV+ OPSCC to reduce treatment‐related morbidity while maintaining a favorable prognosis.

Our institution utilizes neoadjuvant chemotherapy followed by transoral robotic surgery (NAC+S) as a novel de‐escalated treatment strategy for selected HPV+ OPSCC patients. The NAC+S protocol is both systemic escalations combined with locoregional de‐escalation, scaling down the surgery by reducing the margin of primary resection, and reserving radiation for salvage only.^4^ Postoperative radiation was recommended for patients with high clinical staging or the presence of at least 1 unfavorable marker found on surgical pathology, including positive margin, extranodal extension (ENE), and perineural invasion (PNI).

With the advancement of treatment modality and favorable prognosis of OPSCC patients, quality of life has emerged as an important outcome measure in evaluating treatment success.^5^ Among various domains of quality of life, swallowing function is reported to be a top concern among OPSCC patients.^6^ NAC+S has been shown to improve 5‐year disease‐free survival with lower feeding dependence compared to stage‐matched patients undergoing concomitant chemoradiation.^4^ Recently, Lee et al showed that patients undergoing NAC+S had favorable long‐term swallowing function with 78.4% achieving optimal swallowing function 2 years posttreatment as assessed by the MD Anderson Dysphagia Inventory (MDADI) survey.^7^ Our study aims to augment the current understanding by comparing swallowing trends between patients who underwent NAC+S only (NAC+S group) and patients who received further adjuvant radiotherapy in addition to NAC+S (NAC+S+R group) at 3 follow‐up periods after completion of treatment: short‐term (<1 year), middle‐term (1‐3 years), and long‐term (>3 years). We hope that this knowledge will help establish patient expectations, guide education, and plan timely interventions to bring the most satisfying swallowing outcomes for patients undergoing NAC+S as their definitive treatment for HPV+ OPSCC.

## Methods

### Study Population

We performed a cross‐sectional cohort study to evaluate swallowing outcomes among OPSCC patients. All patients who were diagnosed with HPV+ OPSCC, received NAC+S as a primary treatment at a single academic institution between January 2006 to December 2020 and completed the MDADI survey were included in this study. Patients with otherwise unclear information were also excluded from the study. Patient characteristics, tumor, and treatment details were obtained from a retrospective chart review. All patients were staged using the 7th edition American Joint Committee on Cancer (AJCC) guideline to maintain consistency as most patients received treatment prior to the release of the 8th edition guidelines. The study was approved by the George Washington University School of Medicine Institutional Review Board (NCR202928).

### Treatment

Neoadjuvant chemotherapy (NAC) was offered to the patients with HPV+ OPSCC, AJCC 7 stage III (T1N1, T2N1, T3N0, T3N1), and stage IVa (T1N2, T2N2, T3N2), with no evidence of distant metastatic disease. NAC includes 3 cycles of taxane‐based chemotherapy consisting of Docetaxel 75 mg/m^2^ and Cisplatin 75 mg/m^2^ every 3 weeks. Surgery was followed at least 3 weeks after the completion of NAC and was performed using a transoral robotic surgery approach with either unilateral or bilateral neck dissection. Postoperative radiation was recommended for patients with high clinical staging or the presence of at least 1 unfavorable marker found on surgical pathology, including positive margin, ENE, and PNI. In our study, the swallowing function of 2 groups of HPV+ OPSCC patients was compared based on treatment modality: NAC+S versus neoadjuvant chemotherapy+surgery+radiation (NAC+S+R).

### Assessment

MDADI was used to assess patients' swallowing function. MDADI is a 20‐item questionnaire with responses on a 5‐point Likert scale that are converted numerically from 1 (strongly agree) to 5 (strongly disagree). It includes 1 question on global function and 19 additional questions related to the emotional, functional, and physical aspects of swallowing. A total score was calculated, ranging from 20 to 100, with higher scores indicating better swallowing function.

MDADI was administered by multiple methods, including in‐person follow‐up visits, by phone, and/or by email provided from electronic records. Participation in the study was voluntary, and the patients could refuse to complete the questionnaire at any time. As MDADI was given at most of the follow‐up visits as part of the standard of care to monitor for patients' swallowing function, a patient could have multiple MDADI scores. However, MDADI was not administered at every follow‐up visit resulting in inconsistent time points for MDADI completion. Thus, MDADI scores for each patient were divided into 3‐time frames: short‐term, middle‐term, and long‐term. Short‐term is defined as MDADI completed less than 1‐year posttreatment completion. Middle‐term is defined as MDADI completed between 1 and 3 years posttreatment completion. Long‐term is defined as MDADI completed more than 3 years posttreatment completion.

### Statistical Analysis

An independent *T* test and a paired *T* test were performed to compare continuous measurements. The *χ* ^2^ test was performed to compare the distribution of categorical data. The linear regression model was employed to build the model to predict the MDADI scores. Missing data were replaced by series means if necessary. Statistical analysis was performed using SPSS statistical software (version 27.0; SPSS Inc). Statistical significance was established at *p* < .05.

## Results

**Figure 1:**
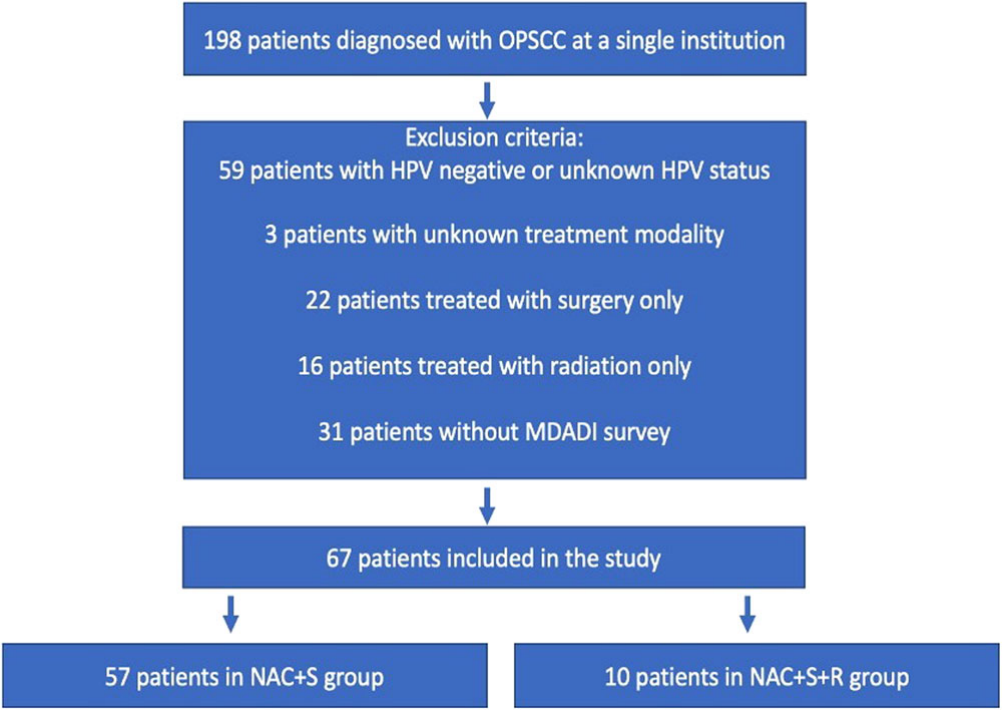
Inclusion flowchart. HPV, human papillomavirus infection; MDADI, MD Anderson Dysphagia Inventory; NAC+S, neoadjuvant chemotherapy and surgery; NAC+S+R, neoadjuvant chemotherapy, surgery, and adjuvant radiotherapy; OPSCC, oropharyngeal squamous cell carcinoma.

A total of 198 patients who were diagnosed with OPSCC at a single academic institution between January 2006 to December 2020 were identified. After excluding 59 patients with HPV negative or unknown HPV status, 3 patients with unknown treatment modality, 22 patients treated with surgery only, and 16 patients treated with radiation only, 98 patients were identified to be diagnosed with HPV+ OPSCC and underwent NAC+S as a primary treatment. Of 98 patients, 75 (76.5%) patients underwent NAC+S only and 23 (23.5%) patients underwent additional adjuvant chemoradiation. Upon excluding 31 patients without completing the MDADI survey, 67 patients were included in the study. The NAC+S group had 57 patients (85.1%), and the NAC+S+R group had 10 patients (14.9%) (Figure 1).

Patient and tumor characteristics were compared between the NAC+S group and the NAC+S+R group in Table 1. The average age of NAC+S was 57.1 years old (range: 45‐77) and NAC+S+R was 59.4 years old (range: 50‐74). There were more males in both groups (NAC+S: “Male” 89.5% [N = 51] vs “Female” 10.5% [N = 6]; NAC+S+R: “Male” 90.0% [N = 9] vs “Female” 10.0% [N = 1]). Similarly, “white” was the most predominant race in both groups (NAC+S: 68.4% [39]; NAC+S+R: 90.0% [9]). There were more nonsmokers and never‐heavy alcohol users in both groups. (NAC+S: “Non‐smoker” 66.7% [38], “Never heavy” 87.7% [50]; NAC+S+R: “Non‐smoker” 50.0% [5], “Never heavy” 70.0% [7]). The primary tumor site was evenly distributed between the base of tongue (BOT) and the tonsil in both groups (NAC+S: “BOT” 52.6% [30], “Tonsil” 47.4% [27]; NAC+S+R: “BOT” 40.0% [4], “Tonsil” 60.0% [6]). When comparing unfavorable pathology, the NAC+S+R group had more patients with positive PNI compared to the NAC+S group (NAC+S vs NAC+S+R: 3.5% [2] vs 20.0% [2]; *p* = .042). The NAC+S group had more patients in T1 and N0 stages compared to the NAC+S+R group (NAC+S vs NAC+S+R: “T1” 16.1% [9] vs 0% [0], *p* = .037; “N0” 14.3% [8] vs 0% [0], *p* = .001). The NAC+S group had more patients with the complete response to NAC+S with a pathology stage of T0N0 compared to the NAC+S+R group (NAC+S vs NAC+S+R: 56.1% [32] vs 10.0 [1]; *p* = .001). The NAC+S+R group had more patients who underwent bilateral neck dissection compared to the NAC+S group (NAC+S vs NAC+S+R: 52.6% [30] vs 90.0% [9]; *p* = .027). There was no difference in postoperative complication between the 2 groups (*p* = .880).

**Table 1:** Patient and Tumor Characteristics Between NAC+S Versus NAC+S+R Group

	NAC+S, N (%)	NAC+S+R, N (%)	*p* value
Total	57 (85.1)	10 (14.9)	
Age, mean (range)	57.1 (45‐77)	59.4 (50‐74)	
Sex			
Male	51 (89.5)	9 (90.0)	.960
Female	6 (10.5)	1 (10.0)	
Race			
White	39 (68.4)	9 (90.0)	.540
African American/Black	10 (17.5)	0 (0)	
Hispanic	1 (1.8)	0 (0)	
Asian	3 (5.3)	0 (0)	
Other	4 (7.0)	1 (10.0)	
Smoking			
Nonsmoker	38 (66.7)	5 (50.0)	.240
Ex‐smoker	16 (28.1)	3 (30.0)	
Current smoker	3 (5.3)	2 (20.0)	
Alcohol use			
Never heavy	50 (87.7)	7 (70.0)	.076
Previous heavy	4 (7.0)	3 (30.0)	
Current heavy	3 (5.3)	0 (0.0)	
Primary tumor site			
Base of tongue	30 (52.6)	4 (40.0)	.461
Tonsil	27 (47.4)	6 (60.0)	
Unfavorable pathology			
Positive margin	6 (10.5)	2 (20.0)	.394
Positive ENE	3 (5.3)	1 (10.0)	.560
Positive PNI	2 (3.5)	2 (20.0)	*.042
T stage			
T1	9 (16.1)	0 (0.0)	*.037
T2	34 (60.7)	7 (70.0)	
T3	12 (21.4)	1 (10.0)	
T4a	1 (1.8)	2 (20.0)	
N stage			
N0	8 (14.3)	0 (0.0)	*.001
N1	16 (28.6)	1 (10.0)	
N2a	6 (10.7)	0 (0.0)	
N2b	23 (41.1)	3 (30.0)	
N2c	3 (5.4)	3 (30.0)	
N3	0 (0)	2 (20.0)	
Overall stage			
I	1 (1.8)	0 (0.0)	.450
II	4 (7.1)	0 (0.0)	
III	17 (30.4)	1 (10.0)	
IVa	33 (57.1)	7 (70.0)	
IVb	2 (3.6)	1 (10.0)	
Path stage			
T0N0	32 (56.1)	1 (10.0)	
T0N1	12 (21.1)	1 (10.0)	
T1N0	7 (12.3)	0 (0)	
T1N1	4 (7)	2 (20.0)	
T1N2a	0 (0)	1 (10.0)	
T1N2c	0 (0)	1 (10.0)	
T2N0	1 (1.8)	0 (0)	*.001
T2N1	0 (0)	1 (10.0)	
T2N2c	0 (0)	1 (10.0)	
T3N0	1 (1.8)	0 (0)	
T3N2a	0 (0)	1 (10.0)	
T4aN0	0 (0)	1 (10.0)	
Neck dissection			
Unilateral	27 (47.4)	1 (10.0)	*.027
Bilateral	30 (52.6)	9 (90.0)	
Postoperative complication			
Chyle leak	3 (5.3)	1 (10.0)	.880
Postoperative bleeding	1 (1.8)	0 (0.0)	
Spinal accessory nerve injury	1 (1.8)	0 (0.0)	

Abbreviations: ENE, extranodal extension; NAC+S, neoadjuvant chemotherapy and surgery; NAC+S+R, neoadjuvant chemotherapy, surgery, and adjuvant radiotherapy; PNI, perineural invasion.

* Represents statistical significance at *p* < .05.

Mean differences in MDADI scores during short‐term, middle‐term, and long‐term follow‐ups were compared between the NAC+S and the NAC+S+R groups (Table 2). Within the NAC+S group, the patients had better MDADI scores in the middle‐term compared to the short‐term (mean difference S‐M = −3.43, *p* = .002), at long‐term compared to short‐term (mean difference S‐L = −6.97, *p* < .001), and at long‐term compared to middle‐term (mean difference M‐L = −3.54, *p* = .043). Similarly, within the NAC+S+R group, the patients had better MDADI scores in the middle‐term compared to the short‐term (mean difference S‐M = −11.18, *p* = .044), better MDADI score in the long‐term compared to short‐term (mean difference S‐L = −20.35, *p* < .001), and better MDADI score at long‐term compared to middle‐term (mean difference M‐L = −9.18, *p* = .026).

**Table 2:** Within Patient MDADI Score During Short‐Term, Middle‐Term, and Long‐Term Follow‐up Between NAC+S and NAC+S+R

	NAC+S					NAC+S+R				
			95% confidence interval for the difference					95% confidence interval for the difference		
Time frame	Mean difference	Std Dev	Lower bound	Upper bound	*p* value	Mean difference	Std Dev	Lower bound	Upper bound	*p* value
Short term‐middle term	−3.43	7.78	−5.50	−1.36	.002*	−11.18	15.11	−21.98	−0.37	.044*
Short term‐long term	−6.97	14.10	−10.71	−3.24	<.001*	−20.35	13.32	−29.88	−10.82	<.001*
Middle term‐long term	−3.54	12.82	−6.97	−0.11	.043*	−9.18	10.94	−17.00	−1.35	.026*

Short term: 3 years; middle term: 1‐3 years; and long term: >3 years.

Abbreviations: NAC+S, neoadjuvant chemotherapy and surgery; NAC+S+R, neoadjuvant chemotherapy, surgery, and adjuvant radiotherapy; Std Dev, standard deviation.

* Represents statistical significance at *p* < .05 using paired *t* test.

**Figure 2:**
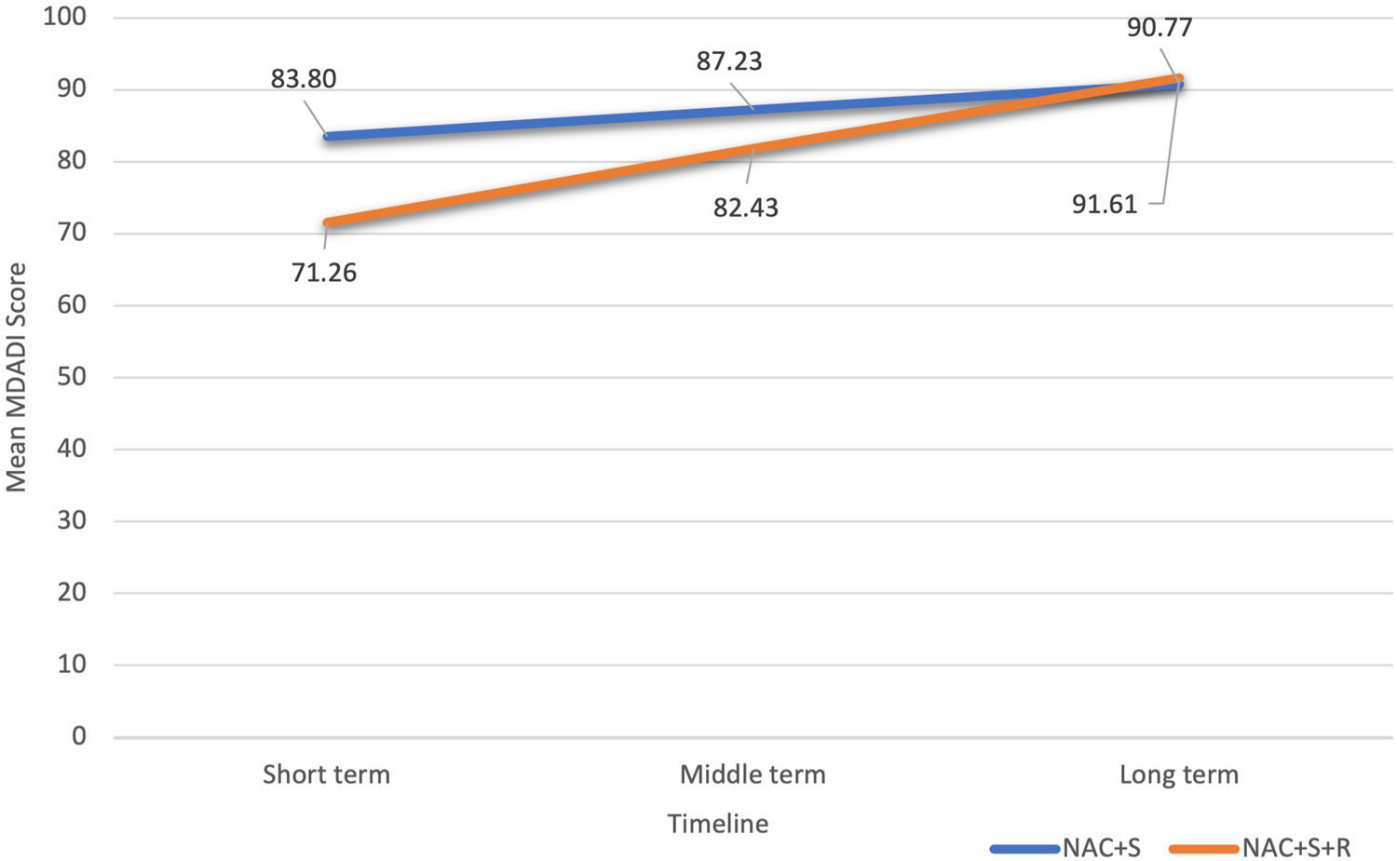
Mean MDADI scores comparison between the NAC+S and NAC+S+R treatment groups. MDADI, MD Anderson Dysphagia Inventory; NAC+S, neoadjuvant chemotherapy and surgery; NAC+S+R, neoadjuvant chemotherapy, surgery, and adjuvant radiotherapy.

MDADI scores between the NAC+S and NAC+S+R groups were compared in the short‐term, middle‐term, and long‐term (Figure 2). Mean MDADI scores for the NAC+S group at short‐term, middle‐term, and long‐term were 83.80 (standard deviation [SD] = 10.04), 87.23 (SD = 8.69), and 90.77 (SD = 10.10), respectively. Mean MDADI scores for the NAC+S+R group at short‐term, middle‐term, and long‐term were 71.26 (14.60), 82.43 (7.75), and 91.61 (6.33), respectively. The NAC+S+R group had a lower MDADI score at short‐term follow‐up compared to the NAC+S+R group (NAC+S vs NAC+S+R: 83.80 vs 71.26; *p* = .001). However, there was no statistical difference in mean MDADI score between 2 groups at middle‐term and long‐term (NAC+S vs NAC+S+R: “Middle‐term” 87.23 vs 82.43, *p* = .107; “Long‐term” 90.77 vs 91.61, *p* = .801). The details of this finding are summarized in Table 3. The linear regression model showed that the NAC+S+R group had a 12.55‐point lower MDADI score compared to the NAC+S in the short term (*p* < .001). However, there were no significant score differences between the 2 groups in the middle term (*p* = .067) (Table 4).

**Table 3:** MDADI Scores at Different Time Frames Between NAC+S Versus NAC+S+R

	NAC+S		NAC+S+R		
Time frame	Mean	Std Dev	Mean	Std Dev	*p* value
Short term	83.80	10.04	71.26	14.60	**.001**
Middle term	87.23	8.69	82.43	7.75	.107
Long term	90.77	10.10	91.61	6.33	.801

Short term <1 year; middle term 1‐3 years; and long term: >3 years.

Bolded are statistical significance at *p* < .05 using an independent *t* test.

Abbreviations: NAC+S, neoadjuvant chemotherapy and surgery; NAC+S+R, Neoadjuvant chemotherapy, surgery, and adjuvant radiotherapy; Std Dev, standard deviation.

**Table 4:** Linear Regression Analysis of Short‐term and Middle‐Term MDADI Scores for NAC+S and NAC+S+R

	Short term					Middle term				
			95% Confidence interval					95% Confidence interval		
	*B*	Standard error	Lower bound	Upper bound	*p* value	*B*	Standard error	Lower bound	Upper bound	*p* value
Intercept	83.80	1.41	81.04	86.56		87.23	1.12	85.04	89.42	
treatment group										
NAC+S+R	−12.55	3.64	−19.69	−5.40	<.001*	−4.80	2.89	−10.47	0.87	.097
NAC+S	0					0				

Short term: <1 year; middle term: 1‐3 years.

Abbreviations: NAC+S, neoadjuvant chemotherapy and surgery; NAC+S+R, neoadjuvant chemotherapy, surgery, and adjuvant radiotherapy.

* Represents statistical significance at *p* < .05.

**Figure 3:**
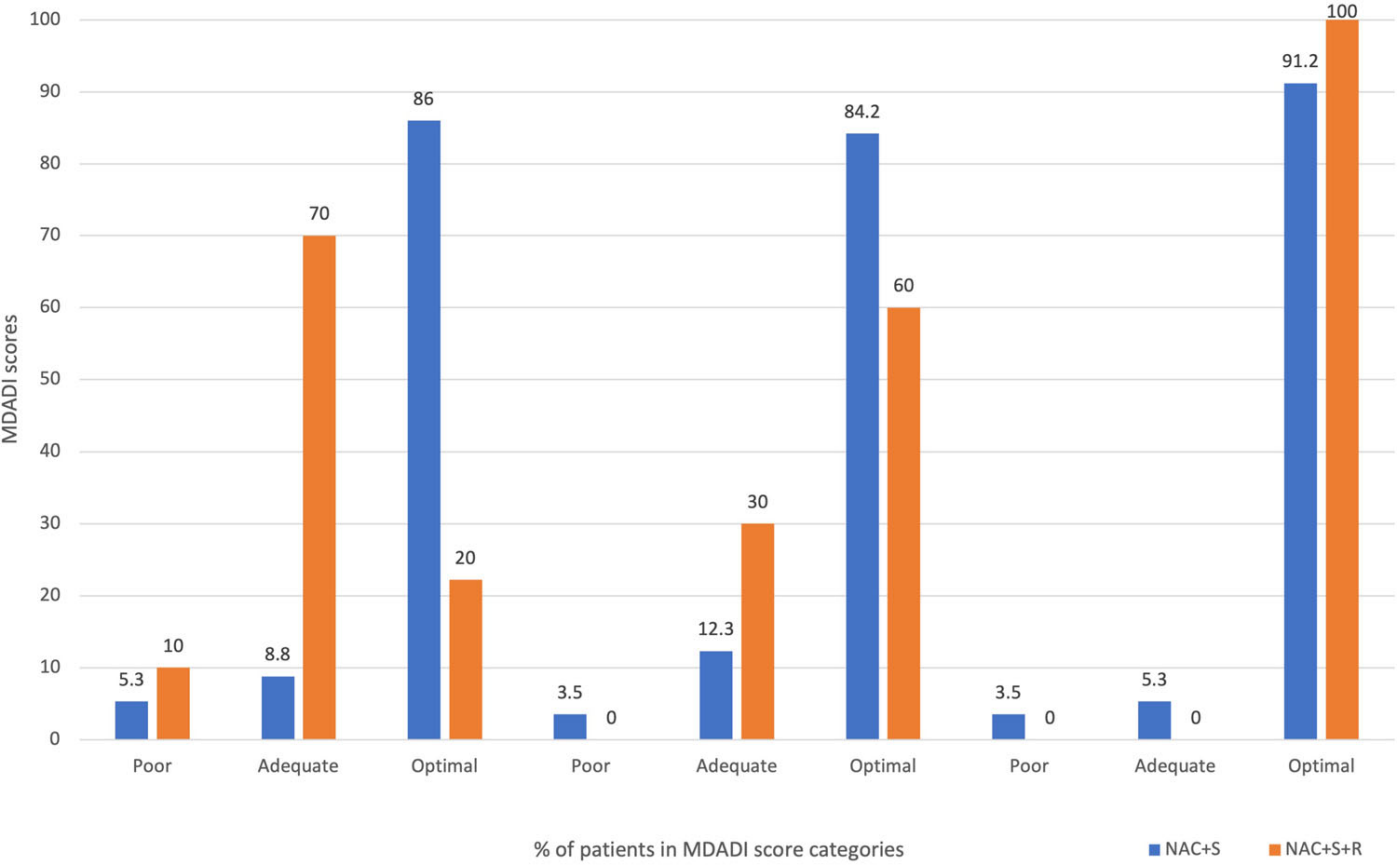
Distribution of patients by MDADI score categories. MDADI, MD Anderson Dysphagia Inventory; NAC+S, neoadjuvant chemotherapy and surgery; NAC+S+R, neoadjuvant chemotherapy, surgery, and adjuvant radiotherapy.

MDADI scores were categorized into poor (<60), adequate (≥60 to <80), and optimal (≥80), and were compared between the NAC+S group and the NAC+S+R group at short‐, middle‐, and long‐term (Table 5). The NAC+S group had more patients in the optimal category compared to the NAC+S+R group in the short term (NAC+S vs NAC+S+R: “Optimal” 86.0% [49] vs 20.0% [2]; *p* < .001). There were no significant differences in the distribution of MDADI score categories between these 2 groups in the middle term and long term. These findings are demonstrated in Figure 3.

**Table 5:** Distribution of MDADI Categories Between NAC+S Group and NAC+S+R Group at Short, Middle, and Long Term

	NAC+S, N (%)	NAC+S+R, N (%)	*p* value
Short term			
Poor	3 (5.3)	1 (10.0)	**<.001**
Adequate	5 (8.8)	7 (70.0)	
Optimal	49 (86.0)	2 (20.0)	
Middle term			
Poor	2 (3.5)	0 (0)	.309
Adequate	7 (12.3)	3 (30.0)	
Optimal	48 (84.2)	6 (60.0)	
Long term			
Poor	2 (3.5)	0 (0)	.623
Adequate	3 (5.3)	0 (0)	
Optimal	52 (91.2)	10 (100.0)	

Short term: <1 year; middle term: 1‐3 years; and long term: >3 years.

MDADI score <60 being “poor,” ≥60 to <80 being “adequate,” and ≥80 being “optimal” swallowing function.

Bolded are statistical significance at *p* < .05 using the *χ* ^2^ test.

Abbreviations: MDADI, MD Anderson Dysphagia Inventory; NAC+S, neoadjuvant chemotherapy and surgery; NAC+S+R, neoadjuvant chemotherapy, surgery, and adjuvant radiotherapy.

## Discussion

Current management for oropharyngeal carcinoma based on National Comprehensive Cancer Network guidelines is either definitive radiotherapy (with or without chemotherapy) or primary surgery (transoral or open, with or without neck dissection) followed by adjuvant radiotherapy as indicated.^8^ The advent of surgical techniques using the transoral robotic approach has emerged as a viable treatment option in the management of OPSCC with high rates of locoregional disease control, survival outcomes, and better functional outcomes.^9^, ^10^ For patients with HPV+ OPSCC, our institution prefers definitive treatment with NAC and transoral robotic surgery to spare patients from the negative consequences associated with radiation.^11^ Nevertheless, a small subset of patients with extensive tumor burden require further adjuvant radiotherapy even after NAC+S treatment. In our study, we aimed to further augment the understanding of swallowing function in the NAC+S cohort by comparing outcomes with those of patients who underwent triple therapy with NAC+S+R in a time‐dependent manner.

Our study found several critical trends in swallowing function in the NAC+S and NAC+S+R treatment groups. First, we found that patients treated with NAC+S had significantly better swallowing function at short‐term follow‐up (<1 year) compared to the patients treated with NAC+S+R. When short‐term MDADI scores are further subclassified, patients treated with the NAC+S were significantly more likely to report optimal swallowing function (MDADI score >80) compared to the patients treated with NAC+S+R (86% vs 20%). This appears consistent with published literature, as other studies also report lower functional outcomes in patients who underwent primary chemoradiotherapy compared to those who underwent TORS with adjuvant therapy. One study reported that the patients treated with TORS and adjuvant therapy scored more than 20 points higher on MDADI assessments compared to patients treated with primary chemoradiotherapy at 12‐month follow‐up.^12^ Similarly, another study showed that patients treated with chemoradiotherapy showed more profound deterioration of swallowing function from baseline in the first 12 months compared to patients treated with transoral laser microsurgery.^10^ Several studies have demonstrated that early postoperative rehabilitation strategies, including tongue resistance exercises, Mendelsohn maneuvers, range of motion exercises, and implementation of speech‐language pathology care improve swallowing function in patients with oropharyngeal carcinoma.^13^, ^14^, ^15^ We suggest additional emphasis placed on such rehabilitation measures in patients who undergo NAC+S+R in order to minimize their early reduction in swallowing function compared to patients who undergo NAC+S.

Second, we found that patients in both groups showed improvement in swallowing function at both middle‐term (1‐3 years) and long‐term (>3 years) follow‐ups compared to the short‐term (<1 year). The swallowing function also improved in the long term (>3 years) compared to the middle term in both treatment groups. This continued improvement in swallowing function beyond 1‐year posttreatment in NAC+S patients is consistent with the reported data in the literature; patients treated with surgical techniques have been shown to have continued improvement after 1 year.^12^, ^16^, ^17^, ^18^ However, our findings of consistent swallowing improvement in patients treated with NAC+S+R counter the concept of “late‐RAD,” which refers to swallowing difficulties caused by long‐term radiation therapy.^19^ In fact, several studies have shown that persistent chemoradiation therapy results in long‐term deterioration of swallowing function even if the ability is initially preserved.^11^, ^20^, ^21^

Similarly, when comparing swallowing function between the NAC+S and the NAC+S+R groups, the significance was only shown at short‐term follow‐up (NAC+S vs NAC+S+R: 83.8 vs 71.26; *p* = .001), but not at middle‐term and long‐term follow‐up. Specifically, we found that long‐term average MDADI scores were nearly identical in patients treated with NAC+S vs those treated with NAC+S+R (90.77 vs 91.70, respectively). We postulate that the persistent improvement seen in our study may be due to multiple causes. First, we believe that NAC results in a reduced extent of resection. Additionally, NAC may result in reduced radiation dosage in patients who require adjuvant radiation therapy, resulting in the preservation of long‐term swallowing function. We believe that usage of NAC results in a de‐escalation of severity at each treatment stage, which may contribute to preserved long‐term swallowing function. Although the definitive explanation and recommendations are limited due to the small sample size in the NAC+S+R group, our results demonstrate the possibility of favorable swallowing function in NAC+S cohorts regardless of adjuvant radiotherapy status. Given the established negative long‐term reduction in swallowing function caused by radiation therapy and the feared development of late‐RAD, initial treatment with NAC+S presents a possible approach to reduce the long‐term worsening in swallowing function in HPV+ OPSCC patients.

There are several limitations to our study. First, we did not capture preoperative MDADI scores. Without a baseline, we did not definitively assess the extent of the postoperative degree and quality of change during the follow‐up period (i.e., the degree of restoration of swallowing function). However, all patients followed a normal pretreatment diet, showing that patients had preserved swallowing function before treatment. This allowed us to conclude that measured MDADI scores during follow‐up were unlikely to be skewed by baseline abnormal swallowing function and further allowed us to use this study as an evaluation of how adjuvant radiation therapy changes MDADI score and swallowing function. Another limitation is the lack of standardization of follow‐up times for MDADI assessment. The timing of survey administration differed between individual patients, which may have resulted in variable distribution within short, middle, and long‐term subcategories. Also, the long‐term follow‐up time set at 3 years may be arbitrary as the extent of dysphagia at 3 years may be different than in later years for some patients. However, several other studies used a long‐term follow‐up point of 2 years and showed stable swallowing function after this time.^11^ Since the NAC+S+R treatment group was small, with 10 patients included in the analysis, additional studies with longer follow‐up times that compare larger treatment groups are warranted to improve the generalizability and validity of the study. Such studies would also allow for higher‐powered analyses and reduce the impact of inherent biases and limitations based on the selection in the comparison groups. Lastly, the nature of the patient‐reported subjective swallowing assessments may be vulnerable to biases and other validity issues, even though they have been widely accepted as reliable and validated.^5^, ^22^

We report several findings that can help establish expectations, guide patient education, and maximize long‐term swallowing function in p16+ OPSCC patients:

1. Both patients treated with NAC+S and NAC+S+R have continued improvement in swallowing function at short (<1 year), medium (1‐3 years), and long (3+) term follow‐ups.
2. Patients treated with NAC+S have significantly better swallowing function at short‐term follow‐up compared to patients treated with NAC+S+R.
3. Swallowing function does not significantly differ at middle‐ and long‐term follow‐up between patients treated with NAC+S and NAC+S+R.

## Conclusion

We found that patients treated with NAC+S have significantly better short‐term swallowing function when compared to patients treated with NAC+S+R. However, long‐term swallowing scores are nearly identical when comparing the 2 treatment groups.

## Author Contributions

**Esther Lee**, concept, design, acquisition, analysis, interpretation of data, drafting of the manuscript, critical revision of the manuscript for important intellectual content, accountability for all aspects of the work; **Hari Magge**, concept, design, acquisition, analysis, interpretation of data, drafting of the manuscript, critical revision of the manuscript for important intellectual content, accountability for all aspects of the work; **Isabel Park**, concept, design, acquisition, analysis, interpretation of data, drafting of the manuscript, critical revision of the manuscript for important intellectual content, accountability for all aspects of the work; **Leyn Shakhtour**, concept, design, acquisition, analysis, interpretation of data, drafting of the manuscript, critical revision of the manuscript for important intellectual content, accountability for all aspects of the work; **Ning‐Wei Li**, concept, design, critical revision of the manuscript for important intellectual content, accountability for all aspects of the work; **Jennifer Schottler**, concept, design, critical revision of the manuscript for important intellectual content, accountability for all aspects of the work; **Arjun S. Joshi**, concept, design, critical revision of the manuscript for important intellectual content, accountability for all aspects of the work; **Punam G. Thakkar**, concept, design, critical revision of the manuscript for important intellectual content, accountability for all aspects of the work; **Joseph F. Goodman**, concept, design, critical revision of the manuscript for important intellectual content, accountability for all aspects of the work.

## Disclosures

### Competing interests

None.

### Funding source

None.
